# Darier's disease: a new paradigm for genetic studies in psychiatric disorders

**DOI:** 10.1590/S1516-31802000000600002

**Published:** 2000-11-01

**Authors:** Ricardo Soares Silva

The recent news about the sequencing of the human genome is still echoing in the lay and scientific media, but the implied questions are already being formulated. Such questions always evoke that archetypal scientist-whose-ambition-knows-no-limits, Dr Victor Frankenstein, and his wish to create life by himself (which he accomplished, although the consequences are that's another story).

But the worst temptation ensnaring us, doctors, is subtler and so more likely to be able to catch us and distort our world-view. It is to try to superpose the two ways of understanding disease, the syndromic-pathological and the biological-genetic, and in the process, to create a monster as deformed as the Creature of Dr Frankenstein (without the articulate and coherent way of expressing itself that is described in the book, I would say…).

The paper on the genetic co-segregation of depression presented in this edition of SPMJ points towards this danger. The idea of studying pairs of diseases, where one of them has a known genetic profile, in order to understand the genetic profile of the other, through their higher or lower association, is clever, well-developed and clearly described, but there is a weak spot in it. Under the generic name of "depression" there are many kinds of affective disorders (bipolar, with or without psychosis, seasonal, etc.), each one with a different pattern of evolution, response to treatment and genetic transmission, and so it is necessary to define *which* depression co-segregates with Darier's syndrome.

And that is the point. The classification of all these disorders under the common name of depression is useful for the comprehension of their common characteristics, internally coherent and a legitimate way of understanding them. All the systems of disease classification in use (ICD-10, MSD-IV), although having imprecisions and controversial points, are based on syndromic thought, clinical observation and the grouping of diseases by their similarity, and they have served us well for a long time.

It is not likely that genetics will defeat the classification systems now in use, for the reasons set out above. However, from the moment that there is a clear definition of what diseases are of genetic origin and what are not, there will then arise an impulse for reformulating the system, separating the two groups without regard for their common clinical characteristics (what will happen to the motto, "clinic rules"?). Against this, I would like to suggest something like MSD-IV, in which diagnosis is expressed along five axes: psychiatric disorders, developmental disorders, systemic concomitant disease, level of relevant stress factors, and level of loss of function –and maybe a sixth axis, genetic factors, with a modifier to indicate whether it refers to axis I, II or III…?

The major advantage of a multi-axial system is that it recognizes the existence of multiple agents (organic, psychic, environmental) acting on the biopsycho-social system we call human and causing disease. This would validate both visions, organicist and psychosocial, and (it is to be hoped) stop their tiresome conflict by satisfying both sides (or, as the Creature said, "Make me happy, Master, and I will be virtuous").

Just one more look at Frankenstein: he admittedly made the Creature big because of the technical difficulties of micro-sutures – and that is the lesson for us. The elegance, conciseness and efficiency of the new classification system will depend on the precision of those who will have the mission of integrate the findings from genetic research with the present medical knowledge. Good luck to them!

